# Observation of odd-parity superconductivity in UTe_2_

**DOI:** 10.1073/pnas.2419734122

**Published:** 2025-03-25

**Authors:** Zixuan Li, Camilla M. Moir, Nathan J. McKee, Eric Lee-Wong, Ryan E. Baumbach, M. Brian Maple, Ying Liu

**Affiliations:** ^a^Department of Physics, Materials Research Institute, The Pennsylvania State University, University Park, PA 16802; ^b^Department of Physics, University of California San Diego, La Jolla, CA 92093; ^c^Department of NanoEngineering, University of California, San Diego, CA 92093; ^d^DC Magnetic Field Facility, National High Magnetic Field Laboratory, Florida State University, Tallahassee, FL 32310; ^e^Department of Physics, Florida State University, Tallahassee, FL 32306

**Keywords:** superconductivity, pairing symmetry, UTe_2_

## Abstract

Symmetry properties of the order parameter are among the most fundamental characteristics of a superconductor. UTe_2_, which was found to feature an exceedingly large upper critical field and striking reentrant behavior at low temperatures, is widely believed to possess a spin-triplet pairing symmetry. However, unambiguous evidence for such a pairing symmetry is still lacking, especially at zero and low magnetic fields. The presence of an inversion crystalline symmetry in UTe_2_ requires that, if it is indeed a spin-triplet superconductor, the order parameter must be of odd parity. We report here phase-sensitive measurements of the symmetry of the orbital part of the order parameter using the Josephson effect. The selection rule in the orientation dependence of the Josephson coupling between In, an *s*-wave superconductor, and UTe_2_ suggests strongly that UTe_2_ possesses the odd-parity pairing state of B_1_*u* symmetry near zero magnetic field, making it a spin-triplet superconductor. We also report the apparent formation of Andreev surface bound states on the (1−10) surface of UTe_2_.

## Significance

The search for spin-triplet superconductors has been pursued for nearly seven decades, yielding to date no universally accepted success. The recently discovered heavy-fermion superconductor UTe_2_ is the latest serious candidate for spin-triplet superconductivity. However, even though properties of UTe_2_ found so far make it difficult to believe that UTe_2_ can be a spin singlet superconductor, unambiguous evidence for spin-triplet pairing is still lacking, especially in zero and low fields. In the work reported here, the orbital part of the superconducting order parameter is probed directly by phase-sensitive measurements. The establishment of the selection rule in the Josephson effect provides strong evidence for odd-parity, and therefore spin-triplet superconductivity in UTe_2_.

The superconducting order parameter (OP), or the wave function of the Cooper pairs in a superconductor, consists of an orbital and a spin part. The search for superconductors featuring an OP different from the spin-singlet, *s*-wave one used in the original Bardeen–Cooper–Schrieffer theory for superconductivity (1) has been an important direction for superconductivity research (2, 3). UTe_2_, a recently discovered heavy fermion superconductor (4, 5) with a superconducting transition temperature (T_*c*_) up to 2.1 K (6), is widely believed to exhibit odd-parity, spin-triplet pairing due to the observation of extremely high and anisotropic upper critical fields. Along the *b* axis, for example, ${\text{H}}_{c_{2},b}$∼ 20 T in the zero temperature limit, which greatly exceeds the Pauli paramagnetic limit for a spin-singlet superconductor with a T_*c*_ of ∼2 K (7, 8). As the field is increased above $H_{c_{2},b}$, spectacular reentrant superconductivity was observed at low temperatures (5). A rich phase diagram featuring novel phases was found as the field strength and orientation are varied. In particular, when the field is tilted toward the *c* axis from the *b* axis, reentrant superconductivity was found unexpectedly within a range of tilting angle and field strength (5). As the field strength increased even further, a metamagnetic field transition into a magnetic field polarized phase was observed (9). The presence of metamagnetism would seem to suggest that the material is near a ferromagnetic instability. On the other hand, neutron scattering studies have revealed that the anisotropic magnetic fluctuations are strong, *k*-dependent, and peaked at incommensurate *k* values (10, 11). These complex magnetic fluctuations can help facilitate non-*s*-wave pairing (12, 13).

However, whether UTe_2_ is indeed a spin-triplet superconductor is yet to be established unambiguously, in particular, in zero and low applied magnetic fields. The absence of the Hebel-Slichter coherence peak in the 1$/T_{1}$ data (14), where 1$/T_{1}$ is the nuclear spin-lattice relaxation rate, only suggests that UTe_2_ is non-*s*-wave. A small drop in the NMR Knight shift seen with the field along the *b* axis (15) does not necessarily mean that the material is a spin-triplet superconductor. Indeed, the very large spin–orbit coupling (SOC) possessed by UTe_2_ makes it a challenging task to interpret results from measurements aimed at obtaining the spin susceptibility. Specifically, a small drop in the Knight shift could be due to the exceedingly large SOC in UTe_2_ as opposed to spin-triplet pairing (16) or complications from electronic structure as seen long ago in V (17). In addition, multiband spin-singlet superconductors are known theoretically to possess an upper critical field that is complicated to calculate (16). The strongly anisotropic and exceedingly large upper critical field of UTe_2_ (7) does not appear to have been evaluated within the context of multiband superconductivity. These considerations provide strong motivation to pursue Josephson effect–based phase-sensitive experiments probing directly the orbital part of the OP which are not subject to the same limitations described above.

**Fig. 1.**
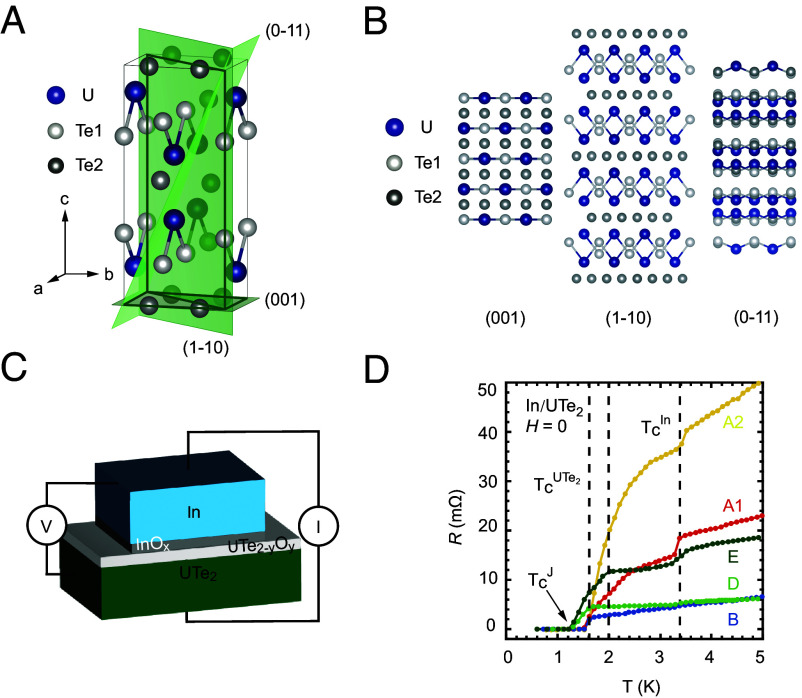
Crystal structure and tunneling junction preparation. (*A*) Crystal structure of UTe_2_ featuring the space group *Immm* (#71) with orthorhombic point group of ${\text{D}}_{2h}$. (*B*) Schematics of crystalline surfaces of (001), (1−10), and (0−11) showing very different atomic arrangements. (*C*) Schematic for an In/UTe_2_ Josephson junction prepared on a flat surface of UTe_2_. (*D*) Curves of sample resistance (*R*) vs. temperature (*T*) in zero magnetic field measured at *I*_*m*_= 200, 100, 500, 100, and 100 μA for Samples A1, A2, B, D, and E, respectively. Drops seen at 1.6 K (2.0 K) and 3.4 K correspond to the T_*c*_’s for UTe_2_ and In, respectively (see *SI Appendix*, Fig. S2*D* for the small drops at 3.4 K in Samples B and D).

For crystalline superconductors with inversion symmetry, the spin and orbital parts of the OP are locked—the superconductor must be either even-parity, spin-singlet or odd-parity, spin-triplet (when subjected to a few conditions such as zero center-of-mass–momentum pairing) (16). According to the Volovik-Gor’kov theory (18), superconductors can be classified by the basis functions of the irreducible representations (irreps) of the symmetry group of the normal state, G×T×U(1), where G is the point-group, T the time-reversal, and U(1) the gauge symmetries, respectively. UTe_2_ has a body-centered orthorhombic crystal structure (Fig. 1*A*) with the point group of $D_{2h}$ (The space group is *Immm*). Various crystalline surfaces with different atomic arrangements may be obtained (Fig. 1*B*). Pairing states allowed by symmetry are listed in Table 1. In the spin-triplet case, the orbital and spin rotations are locked due to the strong SOC.

**Table 1.** Irreducible representations (irreps) and basis functions of pairing states allowed by the normal-state symmetry group for UTe_2_, D_2*h*_ × T × U(1)

Irrep	Basis function	*J*_*s*_ (001)	*J*_*s*_ (1−10)	*J*_*s*_ (0−11)
$\Gamma_{1}^{+}$ or $A_{1g}$	$c_{x}k_{x}^{2}+c_{y}k_{y}^{2}+c_{z}k_{z}^{2}$	≠ 0	≠ 0	≠ 0
$\Gamma_{2}^{+}$ or $B_{1g}$	$ck_{x}k_{y}$	= 0	≠ 0	= 0
$\Gamma_{3}^{+}$ or $B_{2g}$	$ck_{x}k_{z}$	= 0	= 0	= 0
$\Gamma_{4}^{+}$ or $B_{3g}$	$ck_{y}k_{z}$	= 0	= 0	≠ 0
$\Gamma_{1}^{-}$ or $A_{1u}$	$c_{x}\hat{x}k_{x}+c_{y}\hat{y}k_{y}+c_{z}\hat{z}k_{z}$	= 0	= 0	= 0
$\Gamma_{2}^{-}$ or $B_{1u}$	$c_{1}\hat{x}k_{y}+c_{2}\hat{y}k_{x}$	≠ 0	= 0	≠ 0
$\Gamma_{3}^{-}$ or $B_{2u}$	$c_{1}\hat{x}k_{z}+c_{2}\hat{z}k_{x}$	= 0	≠ 0	≠ 0
$\Gamma_{4}^{-}$ or $B_{3u}$	$c_{1}\hat{y}k_{z}+c_{2}\hat{z}k_{y}$	= 0	≠ 0	= 0

The selection rule for the orientation dependence of the Josephson coupling between an *s*-wave and UTe_2_ is shown. *J*_*s*_ is the Josephson current density on the respective junction planes at *T* = 0.

Consider now a Josephson tunnel junction between an *s*-wave and a non-*s*-wave, spin-singlet superconductor. The junction orientation is specified by the unit vector $\hat{n}$ perpendicular to the junction plane. The Josephson current density between two spin-singlet superconductors at a fixed phase difference is given by Millis et al. (19)

[1] $$\begin{array}{c} J_{s}∼{\left\langle \left(\hat{n}\cdot \hat{k}\right)Im\left[\Delta_{s}\Delta^{*}\left(\hat{k}\right)\right]\right\rangle }_{\mathrm{FS}}, \end{array}$$

where $\Delta_{s}$ is the OP of the *s*-wave superconductor, $\Delta (\hat{k})$ is the OP of the non-*s*-wave superconductor, $\hat{k}$ is the quasimomentum vector normalized by the Fermi momentum, and ${\left\langle ...\right\rangle }_{\mathrm{FS}}$ represents an integral over the Fermi surfaces (FS), taking into account the square of the tunneling matrix elements. For a similar junction between an *s*-wave and odd-parity, spin-triplet superconductor, whose Josephson coupling is facilitated by SOC, the corresponding formula is given by refs. 19 and 20

[2] $$\begin{array}{c} J_{s}∼Im{\left\langle \Delta_{s}^{*}\left(\hat{k}\right)\vec{d}\left(\hat{k}\right)\cdot \left(\hat{k}\times \hat{n}\right)\right\rangle }_{\mathrm{FS}}, \end{array}$$

where $\vec{d}\left(\hat{k}\right)$ is the OP of the odd-parity, spin-triplet superconductor. It is important to note that the Josephson junction is assumed to be smooth on the order of the zero-temperature superconducting coherence length in order to derive the two equations listed above using a one-dimensional model (19, 20), keeping in mind that the coherence length is strongly anisotropic in UTe_2_. It was shown that J_*s*_ will vanish in junctions along certain crystalline orientations, known also as the “selection rule” for Josephson coupling. Experimentally, to satisfy the requirement that the surface of the unconventional superconductor on which the Josephson junction is prepared is smooth and the height rms should be smaller than the superconducting coherence length perpendicular to the surface (up to the longest superconducting coherence length in directions parallel with the surface). The selection rule for the Josephson coupling for junctions prepared on the (001), (1−10), and (0−11) surfaces of UTe_2_ is shown in Table 1. Incidentally, the Josephson effect experiment was one of the first to show that Sr_2_RuO_4_ was most likely a spin-triplet superconductor (21) before the more decisive phase-sensitive experiment was performed (16, 22).

The validity of the selection rule in the Josephson coupling also requires that the Josephson coupling is of the first order, which can in practice be inferred from the zero-temperature limit of I_*c*_R_*N*_, where I_*c*_ is the critical current and R_*N*_ is the normal-state junction resistance, respectively. For an all-*s*-wave Josephson junction, it was shown that the upper limit of I_*c*_ at *T*= 0 was given by the Ambegaokar-Baratoff (A-B) formula (23, 24),

[3] $$\begin{array}{c} I_{c}R_{N}=\frac{1}{e}\Delta_{1}K\left({\left[1-{\left(\Delta_{1}/\Delta_{2}\right)}^{2}\right]}^{1/2}\right), \end{array}$$

where $\Delta_{1}$ and $\Delta_{2}$ are the superconducting energy gaps of the two *s*-wave superconductors. Importantly, the I_*c*_R_*N*_ given in Eq. **3** serves as the upper limit—experimentally, the values of I_*c*_R_*N*_ rarely reach the A-B value even for *s*-wave superconductors due to both fundamental (25) and practical issues such as reduction of the superconducting energy gap at the surfaces of the superconducting electrodes.

It was shown previously (26) that the value of I_*c*_R_*N*_ for the first-order Josephson coupling between an *s*-wave and odd-parity superconductor is much smaller than the *s*-wave A-B value calculated using Eq. **3** with the odd-parity superconductor replaced by an *s*-wave one featuring the same superconducting energy gap (26). Should the tunneling be of second order, the I_*c*_R_*N*_ value would be orders of magnitude smaller than the A-B limit.

## 1. Results

We prepared Josephson junctions of In, an *s*-wave superconductor, and UTe_2_ by pressing an In dot onto either a surface of the UTe_2_ crystal formed naturally during the crystal growth or a cleaved one (Fig. 1*C*). The crystals used for samples A1, A2, B, C1, C2, and D are from the same growth batch. X-ray diffraction characterization showed that the crystals were single phase with the expected crystalline structure (*SI Appendix*, Fig. S1). Sample E was prepared on a crystal with higher T_*c*_ grown more recently. The smoothness of the crystal surface is characterized by atomic force microscopy studies (*SI Appendix*, Figs. S4–S6) which suggest that the cleaved surfaces are smoother than the naturally grown ones. But both types of surface are smooth over a length of the order of the zero-temperature superconducting coherence length, which is highly anisotropic. Those expected for the three high-symmetry surfaces are given in *SI Appendix*, Fig. S3. Since both In and UTe_2_ are easily oxidized and the preparation of the junction took place in air, an insulating layer must have formed between the bulk In and UTe_2_, which functions as the tunneling barrier. This tunnel barrier can become too large to allow the Josephson coupling. Because of this, the orientations of the surface were determined by Laue diffraction only after low-temperature measurements were completed. Results of sample resistance (*R*) as a function of temperature, *T*, obtained on five junctions prepared on four single crystals of UTe_2_ are shown in Fig. 1*D*. The presence of zero resistance indicates that the Josephson coupling is finite, with the critical current I_*c*_ larger than the measurement current, I_*m*_. The presence of the Josephson coupling is confirmed by the sensitivity of *R* or I_*c*_ to the application of a magnetic field (*SI Appendix*, Figs. S9 and S10).

**Fig. 2.**
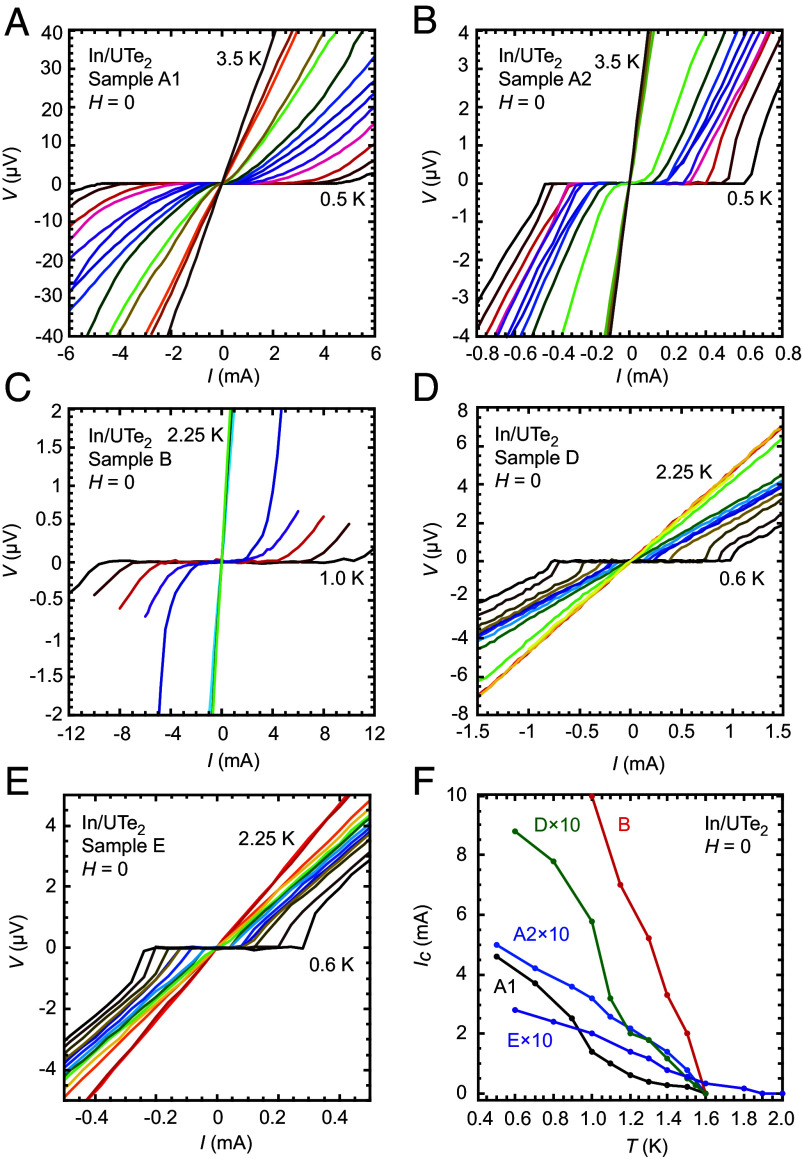
Nonzero Josephson coupling in In/UTe_2_ junctions prepared on (001) and (0−11) surfaces. (*A*–*E*) Voltage (*V*) vs. current (*I*) curves for sample A1 at fixed temperatures *T*= 0.5, 0.7, 0.9, 1.0, 1.1, 1.2, 1.3, 1.4, 1.5, 1.6, 1.7, 2.5, 3.0, and 3.5 K, sample A2 at *T*= 0.5, 0.7, 0.9, 1.0, 1.1, 1.2, 1.3, 1.4, 1.5, 1.6, 2.5, 3.0, and 3.5 K, sample B at *T*= 0.6, 0.7, 0.8, 0.9, 1.0, 1.1, 1.2, 1.3, 1.4, 1.5, 1.6, 1.7, and 2.25 K, sample D at *T*= 0.6, 0.8, 1.0, 1.1, 1.2, 1.3, 1.4, 1.5, 1.55, 1.6, 1.8, 1.9, 2.0, and 2.25 K, Sample E at *T*= 0.6, 0.8, 1.0, 1.1, 1.2, 1.3, 1.4, 1.5, 1.6, 1.8, 1.9, 2.0, and 2.25 K, respectively, showing the presence of finite Josephson coupling. Samples A1 and A2 were prepared on the same crystal surface while sample B was prepared on a different crystal surface, with the same (001) orientation. Samples D and E were prepared on cleaved crystal surfaces of different crystals with (0−11) orientation. The Josephson coupling vanishes at ${\text{T}}_{c}\approx$ 1.6 K for samples A1, A2, B, and D, and for sample E, 2.0 K. (*F*) I_*c*_ (*T*) for samples A1, A2, B, D, and E. The I_*c*_ for samples A2, D, and E are multiplied by 10 for clarity.

Curves of the tunneling current, *I*, vs. the bias voltage, *V*, obtained on samples A1, A2, B, D, and E are shown in Fig. 2*A*–*E*, revealing a finite critical current (I_*c*_) for these junctions. The temperature dependence of I_*c*_ is shown for individual junctions in Fig. 2*F*. The qualitative behavior of I_*c*_(T) is as expected even though its precise functional form is yet to be determined theoretically. A kink is seen in I_*c*_(T) for both samples A1 and D, which may be due to the presence of two parallel Josephson junctions with different values of T_*c*_.

**Fig. 3.**
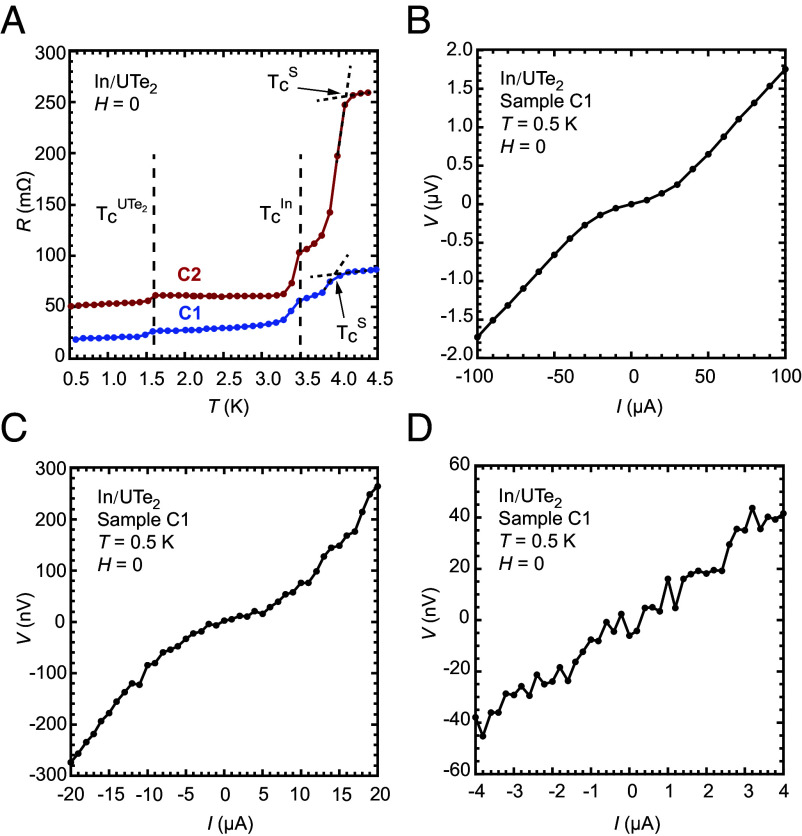
Absence of Josephson coupling in In/UTe_2_ junctions prepared on (1−10) surface. (*A*) R(*T*) curves measured at *I*= 100 μA in zero magnetic fields for both samples C1 and C2. Resistance drops at 1.6 and 3.4 K correspond to the T_*c*_’s for UTe_2_ and In, respectively. The feature seen near 4 K is discussed in the main text. (*B*–*D*) *V*-*I* curves for sample C1 at *T*= 0.5 K measured in decreasing ranges of current. No zero-voltage currents were seen down to the smallest measurement currents.

Two junctions prepared on the same crystal surface, samples C1 and C2, were found to show no zero resistance when their sample resistances were measured at 100 μA down to the lowest temperature (Fig. 3*A*). Both UTe_2_ and In are superconducting as resistance drops were clearly seen at 1.6 K, the T_*c*_ of UTe_2_, and 3.4 K, the T_*c*_ of bulk In. A resistance drop is also seen around 4 K, which is probably the T_*c*_ of In under strain (27, 28), which can come from pressing the In dots onto the crystal and the thermal contraction. *I* − *V* curves obtained from sample C1 at 0.5 K shown in Fig. 3*A*–*C* confirm the absence of supercurrents. To ensure that we do not miss very small supercurrents due to the range of I_*m*_, we systematically reduced the range of current over which the *I* − *V* curves were measured. It is evident that a Josephson current on the order of 1 μA or larger would have been detected. The same behavior was found in sample C_2_ (*SI Appendix*, Fig. S11 *A* and *B*).

As we discussed in *SI Appendix*, atomic force microscopy studies indicate that a naturally formed crystal surface of UTe_2_ has a height rms less than the shortest zero-temperature superconducting coherence length (2.4 nm) for UTe_2_, suggesting that the absence of Josephson coupling is unlikely due to the roughness of the crystal surface. This raises the question of whether the absence of Josephson coupling could be due to a large value of R_*N*_, which, according to Eq. **3**, would make I_*c*_ small. The values of I_*c*_ for Josephson junctions between an *s*-wave and an odd-parity superconductor are expected to be correlated with the R_*N*_ just as all *s*-wave Josephson junctions. The ratios between the experimental value of I_*c*_R_*N*_ to the *s*-wave A-B limit are seen in Table 2 to vary greatly, which could be due to the fact that the gap on surface is reduced from that in the bulk. However, the values of R_*N*_ for samples C1 and C2 would still allow an I_*c*_ value at least one or two orders of magnitude higher than what can be detected easily for the smallest range of I_*m*_ even using the lowest ratio shown in Table 2 for samples in which a finite Josephson coupling was observed. The absence of the Josephson coupling must therefore be due to intrinsic, symmetry-related reasons.

**Table 2.** Summary of parameters for all samples used in the present study

Sample	${\text{T}}_{c}^{R=0}$	${\text{T}}_{c}^{\text{onsite}}$	${\text{R}}_{N}\left(m\Omega \right)$	I_*c*_(*T*_*min*_)(mA)	I_*c*_(*T*_*min*_)R_*N*_ (mV)	I_*c*_(T_min_)R_*N*_ /${{\text{(I}}_{c}{\text{R}}_{N})}_{T=0}^{A-B}$
A1	1.5	1.6	19	4.6	0.087	15%
A2	1.5	1.6	40	0.6	0.024	4%
B	1.5	1.6	5.4	10.0	0.054	9%
D	1.2	1.6	4.7	1.08	0.005	1%
E	1.2	2.0	12	0.26	0.003	0.6%
C1			84	0	0	N/A
C2			253	0	0	N/A

${\text{T}}_{c}^{R=0}$ is the critical temperature for the junction while ${\text{T}}_{c}^{\text{onsite}}$ is for the onset critical temperature. ${{\text{(I}}_{c}{\text{R}}_{N})}_{T=0}^{A-B}$ is the Ambegaokar-Baratoff (A-B) limit calculated assuming that the two superconductors are both *s*-wave.

## 2. Discussion

The orientations of the junction planes were determined using Laue diffraction (*SI Appendix*, Fig. S7*A*) after all low-temperature measurements were completed to minimize exposure to air that tends to increase the oxide layer thickness. Here, the entire surface on which the junction was (or junctions were) made was imaged for each crystal because of the relative sizes of the X-ray beam and the crystal. In *SI Appendix*, Fig. S7 *B*–*D*, we presented Laue images of all three surfaces on which our samples were prepared. The symmetry analysis and simulations for determining the surface orientation suggest that samples A1, A2, and B were prepared on the (001) surface, which happens to be a cleavage plane reported previously (29). Samples D and E were prepared on the (0−11) surface obtained by cleaving a single crystal (30). The orientation of the surface on which samples C1 and C2 were prepared is the (1−10) surface.

The presence of Josephson coupling on (001) and (0−11) surfaces and the absence of it on (1−10) suggest that, among all symmetry-allowed pairing states, the symmetry of the OP adopted in UTe_2_ must be $\Gamma_{2}^{-}$, or ${\text{B}}_{1u}$ (Table 1). Incidentally, it was suggested previously that UTe_2_ may feature an “accidental degeneracy” (30), in which two symmetry-allowed pairing channels happen to have very similar pairing interaction strengths, resulting in the coexistence of two pairing states. In the present case, our selection rule results would be consistent with coexistence of $\Gamma_{2}^{-}$ and another symmetry-allowed pairing state with zero Josephson coupling for the (1−10) surface. Inspection of Table 1 suggests that a pairing state mixing $\Gamma_{1}^{-}$ and $\Gamma_{2}^{-}$ is the only option given that the existence of a pairing state mixing the odd and even parity (spin-triplet and singlet) in UTe_2_ is prohibited by the inversion symmetry (31). Experiments seeking time reversal symmetry breaking carried out recently indicate that the time-reversal symmetry is not broken in the superconducting state of UTe_2_ (32, 33), which seems to suggest that the accidental degeneracy is no longer needed.

The identification of $\Gamma_{2}^{-}$ as the pairing state adopted by UTe_2_ has strong implications on the superconducting properties of UTe_2_. To begin with, the gap function for $\Gamma_{2}^{-}$ has point nodes only at $\vec{k}$ = (0,0,k_*z*_), which means that the superconducting state would be fully gapped if the FS is not a three-dimensional (3D) one. Experimentally, ARPES (29), quantum oscillation measurements (34, 35) and the observation of quantum interference on UTe_2_ (36) suggest that the FS in UTe_2_ is quasi-2D consisting of no “north and south poles.” The observation of a 3D FS in UTe_2_ was also reported (37). On the other hand, single-particle tunneling (30), specific heat (7) and penetration depth (38) measurements revealed the presence of a substantial density of states when samples are superconducting down to the lowest temperatures. A similar situation was encountered in Sr_2_RuO_4_, whose FS is 2D and all symmetry-allowed pairing states are fully gapped. This discrepancy was discussed in the picture of band-dependent superconductivity (16) in which the superconducting energy gap was particularly small in at least one band passing through the FS. The origin of the density of states seen in the superconducting state of UTe_2_ remains to be understood.

**Fig. 4.**
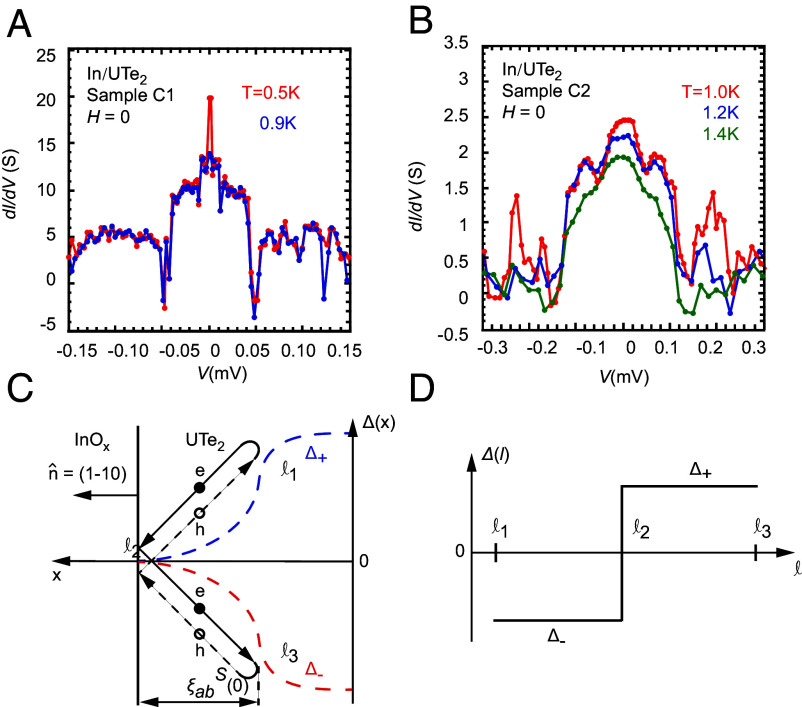
Quasiparticle tunneling spectra. (*A*) d*I*/d*V* vs. *V* for sample C1 at *T* = 0.5 and 0.9 K with the normal-state contribution subtracted (the spectrum obtained at 2.0 K is treated as the normal-state contribution). (*B*) d*I*/d*V* vs. *V* for sample C2 at *T*= 1.0, 1.2, 1.4 K with normal-state contribution obtained at 1.6 K subtracted. (*C*) Schematic illustrating the formation of Andreev surface bound states (ASBSs) in which an electron is Andreev reflected as a hole and reflected off the surface of the crystal (remaining as a hole) before being Andreev reflected again to emerge as an electron. Here, $\Delta_{+}\left(x\right)$ and $\Delta_{-}\left(x\right)$ are OPs for electrons and holes traveling in opposite directions and $\Delta_{+}=\Delta_{-}=0$ at *l*_2_. The superconducting coherence length $\xi_{\mathrm{ab}}^{S}\left(0\right)$ is indicated. (*D*) Schematic illustrating the sign change of the OP along the semiclassical trajectory. The superconducting energy gap vanishes at the surface where the OP changes sign.

In Fig. 4 *A* and *B*, quasiparticle tunneling spectra, d*I*/d*V* vs. *V* curves, obtained from samples C1 and C2 are shown. The relatively high junction resistance allows the *I*-*V* characteristic measurements to be performed up to reasonable bias voltage without heating the samples. The low junction resistance of other samples does not allow the tunneling spectrum to be collected properly. Interestingly, a conductance peak is seen at low bias voltages. It was proposed previously that the formation of zero-energy Andreev surface bound states (ASBSs) lead to a peak near zero bias voltages in the tunneling spectrum in high-T_*c*_ cuprates featuring a pairing state of $\Gamma_{3}^{+}$, or ${\text{B}}_{1g}$ (39, 40), in which the ASBSs were found on the (110) surface because a sign change in the OP is encountered (41). Consider now the quasiparticle motion near the (1−10) surface of UTe_2_. As shown in Fig. 4*C*, if the pairing state is indeed $\Gamma_{2}^{-}$, an electron-like quasiparticle with its energy lower than the bulk gap reflected off the surface (junction plane) will be Andreev reflected as it approaches the interior of the crystal (when its energy matches the position-dependent gap). The Andreev reflected hole-like quasiparticle will be reflected off the surface and then Andreev reflected again, forming a closed-loop semiclassical trajectory at this particular incident/reflection angle. Importantly, the superconducting OP along this semiclassical trajectory will change sign at the surface (Fig. 4 *C* and *D*), leading to the formation of the ASBSs similar to high-T_*c*_ cuprates. It should be noted, however, that a conductance peak at low bias voltages was also seen above the T_*c*_ of UTe_2_ from Samples C1 and C2 (*SI Appendix*, Fig. S11 *C* and *D*), which is below the T_*c*_ of In. Such a conductance peak at low bias was found previously when the superconductor is in the polycrystalline form (42, 43). Even though the current finding is consistent with these previous studies as the In used here is also polycrystalline, more theoretical analysis will be required to understand the physical origin of the observed tunneling features more fully.

## 3. Conclusion

In summary, a finite Josephson coupling between the *s*-wave superconductor In and UTe_2_ was observed on junctions prepared on (001) and (0−11) surfaces but not on the (1−10) surface. This selection-rule result suggests strongly that the pairing symmetry in UTe_2_ at zero and low magnetic fields is that of $\Gamma_{2}^{-}$, or, less likely, a mixture of $\Gamma_{1}^{-}$ and $\Gamma_{2}^{-}$, ruling out other symmetry-allowed spin-triplet and all spin-singlet pairing states. We also observed a feature in the quasiparticle tunneling spectra that we attribute to the formation of ASBSs on the (1−10) surface of UTe_2_. These results provide a strong foundation for understanding other superconducting properties of UTe_2_ including the mechanism of superconductivity. Whether an applied magnetic field, especially a field as high as 20 T, will alter the pairing symmetry in this exotic superconductor remains to be determined.

## 4. Materials and Methods

All single crystals of UTe_2_ used in the present studies were grown at the University of California San Diego using the chemical vapor transport method. Uranium and tellurium in a 2:3 atomic ratio were added to a quartz tube with a mass of iodine equal to 4 mg/cm^3^ relative to the size of the quartz tube and sealed in argon. For the crystals with lower T_*c*_ values (T_*c*_= 1.6 K), the tubes were heated in a gradient of 1,060 °C to 1,000 °C for two weeks, then allowed to cool to room temperature naturally. The crystals with higher T_*c*_ values (T_*c*_= 2.0 K) were heated in a gradient from 830 °C to 710 °C over four weeks and then allowed to cool naturally to room temperature. X-ray diffraction characterization showed that the crystals were single phase with expected crystalline structure (*SI Appendix*, Fig. S1). Selected crystals with a large flat surface or surfaces (up to 1 to 2 mm) were sealed in a glass tube filled with Ar gas after growth and shipped to Penn State. Josephson junctions of In/UTe_2_ were prepared by pressing an In dot of roughly 200 μm in diameter onto a selected surface immediately after taking the crystal out of the sealed glass tube. The orientation of the surface was determined by Laue back reflection after all low-temperature measurements were completed. Among the samples measured in this study, samples A1 and A2 were prepared on the same crystal surface, as were samples C1 and C2. Only sample E was prepared on a crystal with the higher T_*c*_. Electrical leads to UTe_2_ crystals were also prepared using pressed In dots. All samples were mounted to the sample stage of a one-shot He-3 refrigerator and pumped out within a few hours. The He-3 refrigerator is equipped with a superconducting magnet and can reach a based temperature around 0.4 K. Low-temperature transport measurements were carried out in D.C. mode with a Keithley 2,400 as the current source and Keithley 6,182 as the nanovoltmeter. All leads entering the sample space were filtered by RF filters with 15 dB insertion loss at 1 MHz.

## Supplementary Material

Appendix 01 (PDF)

## Data Availability

All data obtained in this study are included in the manuscript and *SI Appendix*, and are available in the ScholarSphere Digital Repository (44).
