# Supplementary material for: Observation of odd-parity superconductivity in UTe2
Source: Proc Natl Acad Sci U S A. 2025 Mar 25;122(13):e2419734122. doi: 10.1073/pnas.2419734122 (PMC12002285; doi:10.1073/pnas.2419734122)
Supplement: Supplementary file 1 — Appendix 01 (PDF) [file pnas.2419734122.sapp.pdf]

Supporting Information for

Supplementary Materials for
Observation of odd-parity superconductivity in UTe_2

Zixuan Li, Camilla M. Moir, Nathan J. McKee, Eric Lee-Wong, Ryan E. Baumbach, M. Brian Maple, Ying Liu.

M. Brian Maple, Ying Liu.
E-mail: mbmaple@ucsd.edu (M.B.M.); yxl15@psu.edu (Y.L.)

This PDF file includes:

Supporting text
Figs. S1 to S11
SI References

Supporting Information Text

Additional information provided below includes the sizes of single crystals of UTe_2 used in the present study, the X-ray diffraction studies of the crystals, the Laue imaging used for the orientation of the surfaces on which the Josephson junctions were prepared, the atomic force microscopy (AFM) images of cleaved plane (0-11) and natural grown surface, low temperature measurements of Samples B, C1 and C2, the response of Josephson junction to magnetic field, and the quasiparticle tunneling spectra.

1. UTe_2 single crystals

To ensure that no impurity phases are present in the single crystals used in the present study, we crushed single crystals grown in the same batch and performed powder X-ray diffraction studies of them, followed by careful analysis to ensure all peaks detected belong to UTe_2 (see fig. S1).

Crystals on which the tunnel junctions were prepared are large in size, possessing at least one flat surface (see Figs. S2A-C). The temperature dependence of the junction resistance exhibits a feature when either In or UTe_2 becomes superconducting. In the case of Sample B, the feature is small (Fig. S2D).

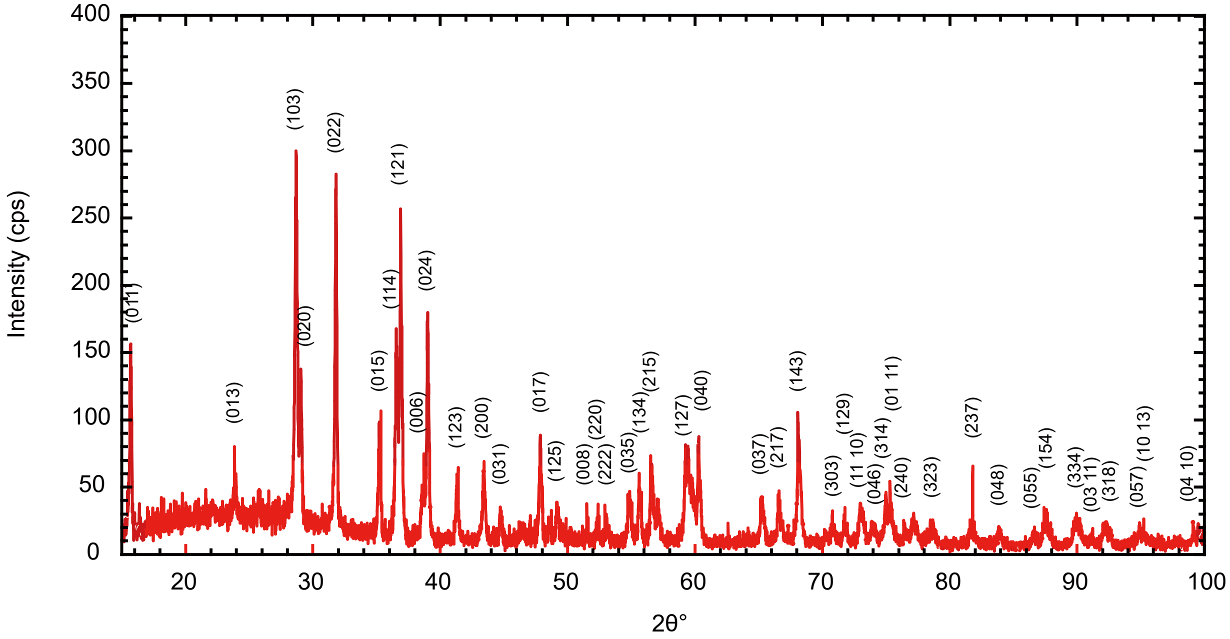

Fig. S1. X-ray diffraction X-ray diffraction data on UTe_2 crystals from the same growth batch as those used in the present experiment. Data were compared against a reference spectrum from the Inorganic Crystal Structure Database (ICSD) (1). All peaks correlate with ICSD UTe_2 reference data with no impurity peaks detected. The small amorphous background at lower angles is due to the quartz powder holder.

Samples A1, A2, B, C and D were prepared on crystals of UTe_2 with a T_c of 1.6 K and sample E on one with a T_c of 2.0 K. The different values of T_c should be due to the level of disorder (2–4), which is known to have strong consequences in certain properties of a superconductor, such as the value of T_c and the residual specific heat below T_c, especially when the superconductor is unconventional. For the detection of the symmetry of the orbital part of the superconducting order parameter reported in the current work, the conclusion we draw from the selection rule result does not depend on the specific value of the Josephson coupling. What matters is whether the Josephson coupling along a specific orientation is zero or nonzero. The level of disorder in the crystal may affect the strength of the Josephson coupling but not whether it is zero or nonzero as long as the disorder does not suppress the T_c too much. The use of crystals of UTe_2 with a T_c value of 1.6 or 2 K will not affect the conclusion on the pairing symmetry of UTe_2.

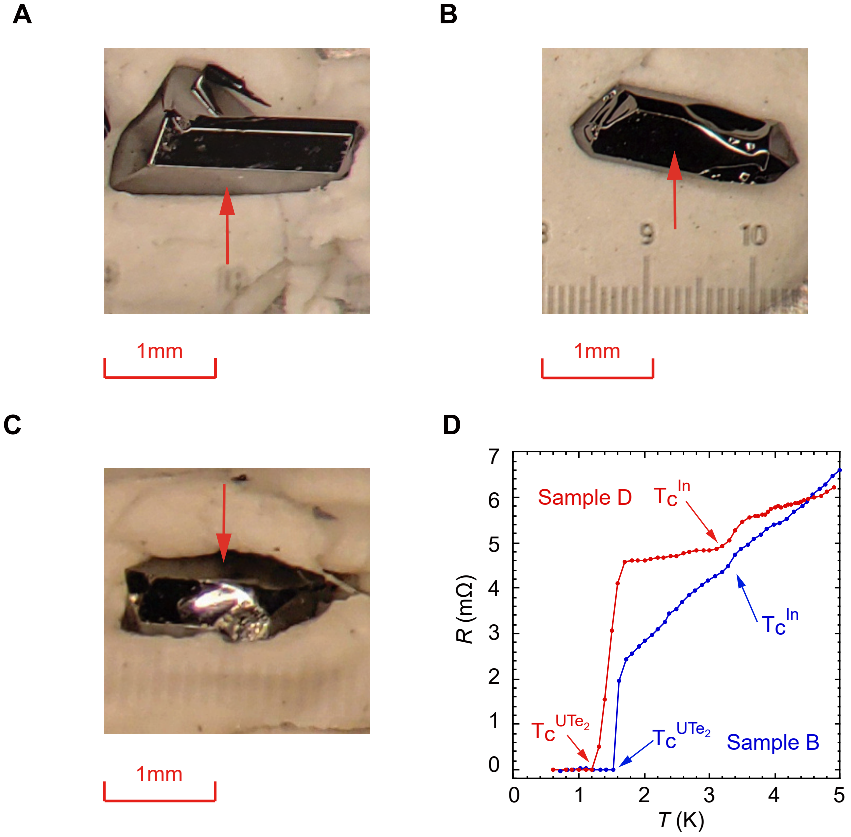

Fig. S2. Single crystals used in this study (A-C) Optical images of the crystals used in the present experiment. The surfaces on which the In/UTe_2 junctions were prepared are indicated. The scale bars are also included. (D) Junction resistance (R) *vs.* temperature (T) curves in zero magnetic fields for samples B and D. Resistance drops at the superconducting transition temperatures for UTe_2 and In are indicated.

2. Smoothness of the crystal surface for junction preparation

As pointed out in the main text, the surface of the UTe_2 crystal on which the Josephson junctions are prepared must be smooth up to the zero-temperature superconducting coherence length. An important question to ask is how smooth is smooth for this purpose. Qualitatively, when the root mean square (RMS) of the height variation is negligible in comparison with the size of a Cooper pair then the surface should be smooth enough. The complication is that the coherence length for UTe_2 is strongly anisotropic even in the bulk. The superconducting coherence length (ξ_a, ξ_b and ξ_c for a, b and c axes) can be calculated by the upper critical fields obtained when the field is applied along the three principal crystal symmetry axes,

$$H_{c2\|a} = \frac{\Phi_0}{2\pi\xi_b\xi_c},$$

$$H_{c2\|b} = \frac{\Phi_0}{2\pi\xi_c\xi_a},$$

$$H_{c2\|c} = \frac{\Phi_0}{2\pi\xi_a\xi_b},$$

The anisotropy leads to an oval shaped normal core of the Abrikosov vortex, as shown schematically in the figure below. On a surface, the coherence length may be even more anisotropic than in the bulk, with both the out-of- and in-plane coherence lengths being affected, which does not seem to have been calculated for a surface with arbitrary orientation. To proceed, it seems reasonable to assume that the out-of-plane coherence length is longer than the shortest coherence length (2.4 nm) and the longest in-plane coherence length is shorter than the longest coherence length (13 nm). Consequently, the surface is smooth if the RMS of the height variation is below 2.4 nm.

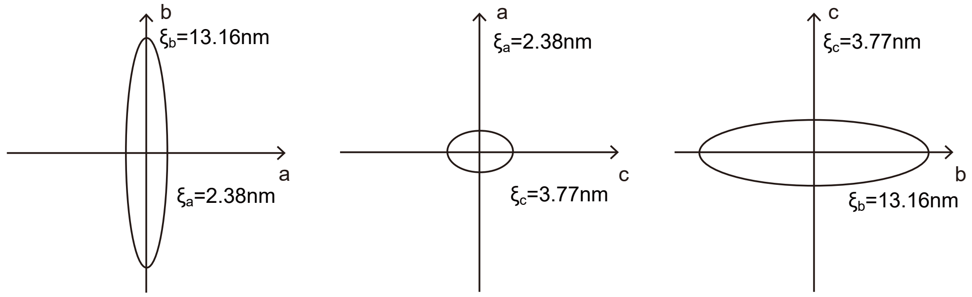

Fig. S3. Schematics of the vortex cores along the three crystalline symmetry axes. The upper critical fields along each axis used for calculation are $H_{c2\|a} = 6T$, $H_{c2\|b} = 35T$ and $H_{c2\|c} = 10T$.

AFM imaging was performed on both the cleaved and naturally grown surfaces with no junctions prepared. Since UTe_2 is an air-sensitive material, AFM studies could not be performed before junction preparation as the long exposure to the air will render the surface unusable for junction preparation. Indeed, it is also not possible that the surfaces on which the junctions were prepared will not be altered after all low-temperature measurements are completed and the pressed In is removed. All crystals used in AFM studies were from the same growth batch. Visually, the surfaces chosen appear the same as other surfaces selected for the preparation of the junctions.

The AFM studies carried out on both the cleaved and naturally grown surfaces of UTe_2 suggest that the junction planes should be sufficiently smooth to ensure that eqs. 1 and 2 in the main text will hold, allowing the use of the selection rule in Josephson coupling to determine the symmetry of the superconducting order parameter.

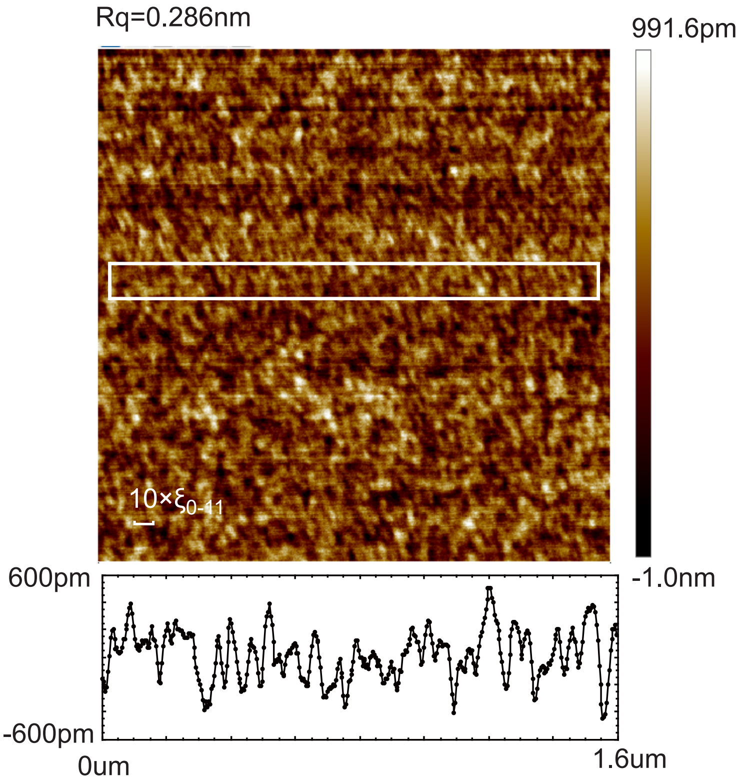

Fig. S4. AFM image of cleaved (0-11) surface of UTe_2 without the presence of steps. The scanning area is $1.6\mu m \times 1.6\mu m$. A scale bar of ten times the largest superconducting coherence length is shown.

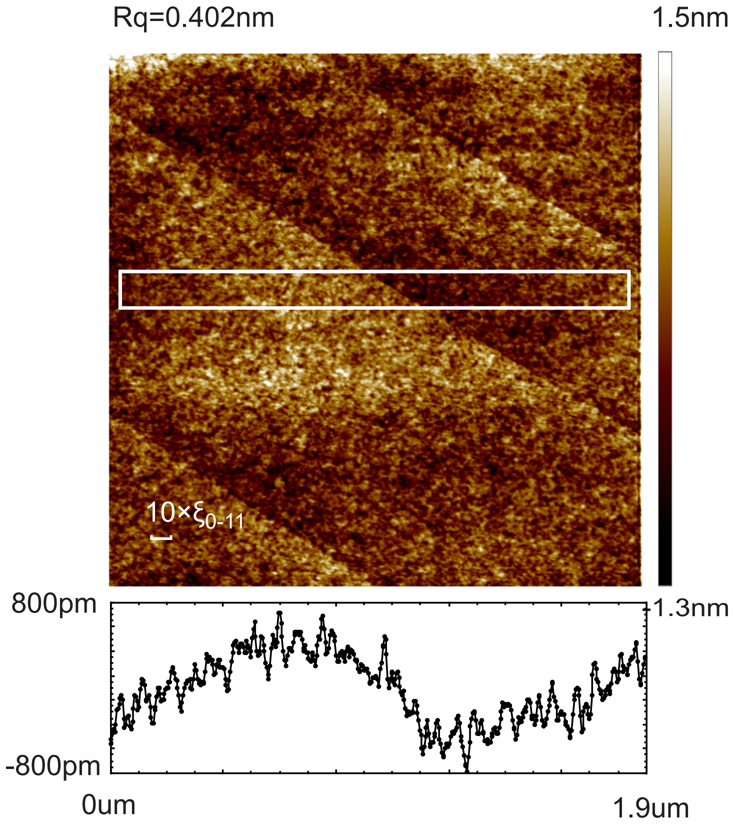

Fig. S5. AFM image of cleaved (0-11) surface of UTe_2 with the presence of steps. The scanning area is 1.9μm×1.9μm. Steps can be seen in this scanning area with a step height roughly 1 nm. A scale bar of ten times the largest superconducting coherence length is shown.

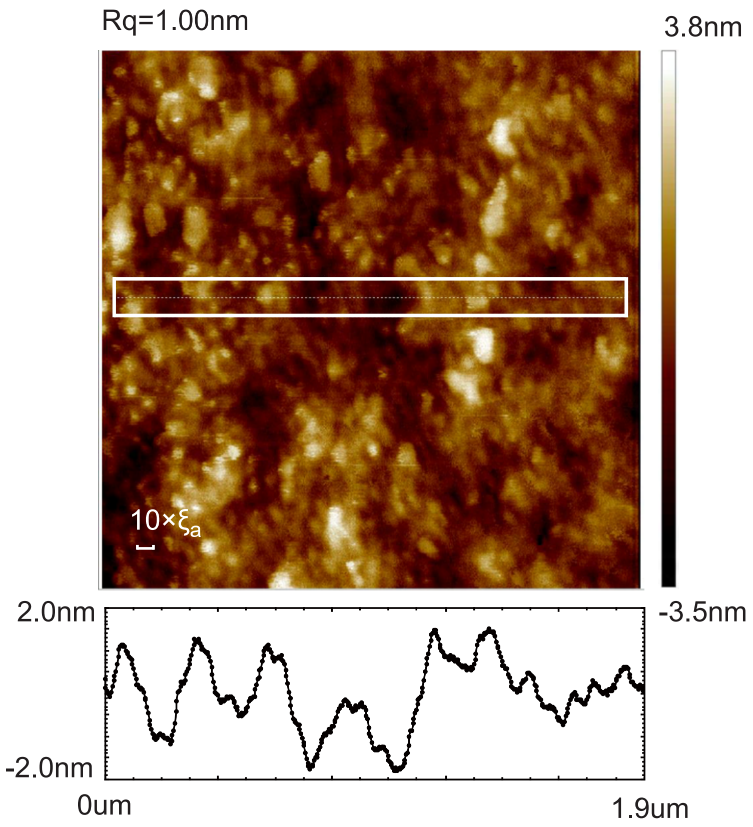

Fig. S6. AFM image of naturally grown surface of UTe_2. The scanning area is 1.9μm×1.9μm. A scale bar of ten times the largest superconducting coherence length is shown.

To ensure that the surface in Fig. S6 represents other naturally grown surfaces on which the junctions were prepared, the crystal for the AFM studies was selected from the same growth batch from which crystals for samples A1, A2, B, C1, and C2 were taken, and the crystal surface appears the same as other naturally grown surfaces chosen to prepare the junctions under the optical microscope. It seems reasonable to assume that all naturally formed surfaces from the same growth batch possess similar smoothness, namely, the surface shown in Fig. S6 has smoothness comparable to those of samples A1, A2, B, C1 and C2.

3. Laue imaging determination of the surface orientation.

Since the selection rule of the Josephson coupling is about the orientation dependence of the Josephson coupling between an s-wave superconductor and UTe_2, the determination of the surface orientation is important. Surface orientation can be found most effectively by Laue diffraction imaging coupled with modeling on the positions of the symmetry allowed diffraction spots. The method and the results are given in fig. S7 and fig. S8.

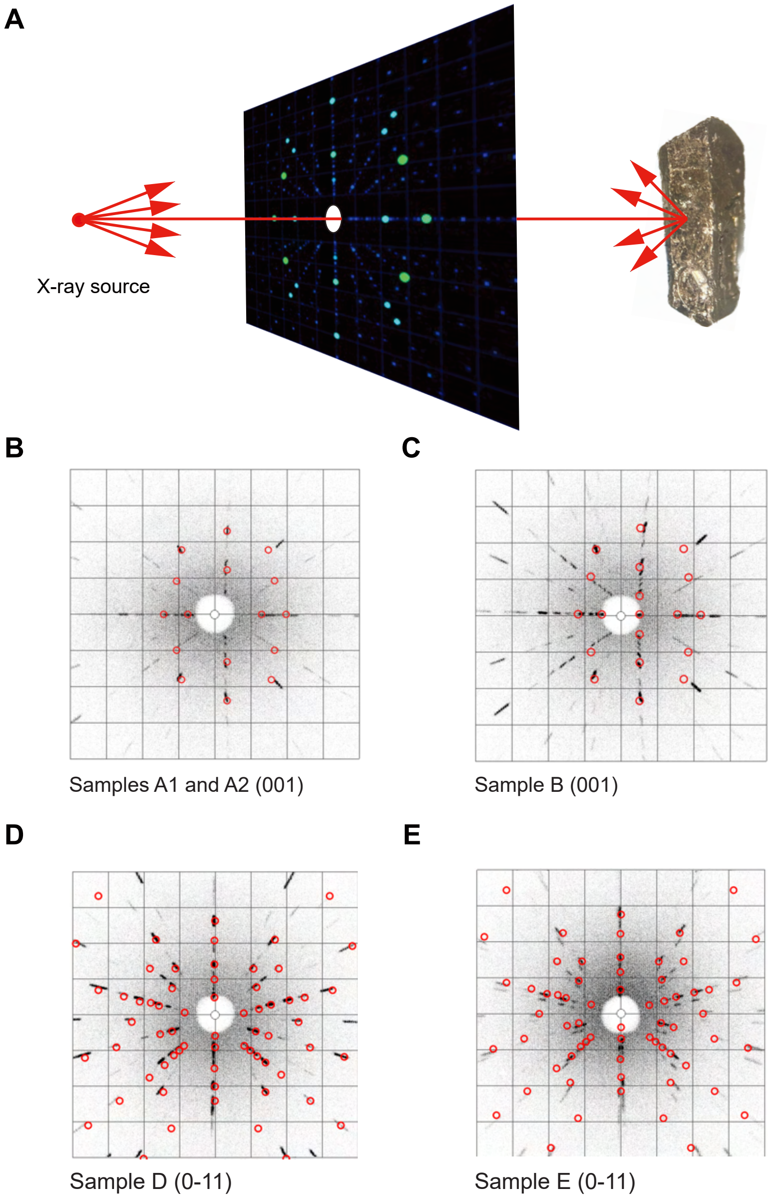

Fig. S7. Laue imaging and modeling. (A) Schematic for Laue imaging method used in the current experiment to determine the orientation of the crystal surface. (B-E) Laue images for crystals A, B, D, E on which various samples were prepared. The modeling of Laue patterns with red circle dots indicating the ideal spots positions. The modeling suggests the surfaces of samples A1, A2 and B are (001) surfaces while for samples D and E are prepared on (0-11) surface.

A crystal surface was selected for the preparation of Josephson junctions based on the size of the surface and its surface smoothness under an optical microscope inspection. However, the orientation of the surface is not known until after all low-temperature measurements are completed. For samples A1, A2 and B, the surface is (001) as indicated by the pattern of nearly four-fold symmetry expected by the closeness of lattice constants a and b. For samples D and E, the surfaces were obtained by cleaving. The (0-11) designation obtained from the modeling is consistent with literature - almost all cleaved surfaces reported thus far are (0-11), as discussed above, featuring a two-fold symmetry. A small offset between the normal vector of the sample surface and the X-ray beam while performing Laue imaging assumed. The symmetry and major spots are seen to match well with the simulation.

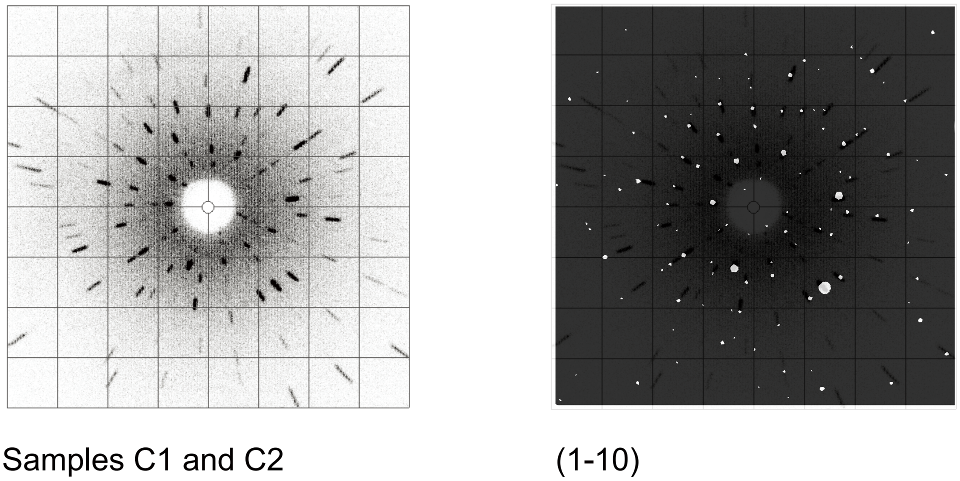

Fig. S8. Laue imaging of crystal C. Left: The Laue pattern obtained on sample C. Right: The modeling of Laue patterns. The white dots indicate the ideal spot positions for the (1-10) orientation, matching well with the black dots from the data. The modeling suggests that samples C1 and C2 are prepared on the (1-10) surface.

For samples C1 and C2, the junctions were prepared on the same naturally formed surface. We began our modeling work by narrowing down the possibilities by going though the Laue images expected from all high symmetry planes. Most spots in the Laue image match the simulation of (1-10). A small deviation from the designated lattice plane that supports a nonzero Josephson coupling will lead to a reduction factor containing the sine and/or cosine of the angle of the deviation, which will not change the Josephson current significantly and change the conclusion on the selection rule result. A deviation from the designated lattice plane which supports zero Josephson coupling may in principle lead to a nonzero Josephson coupling, which would be detected in the measurements. The observation of the selection rule of the Josephson coupling is thus robust against a small deviation of the junction plane from the lattice plane designated by the Laue imaging even though the fits are not perfect.

In principle, magnetic impurities on the surface will also affect the Josephson coupling. Junctions A1, A2, B, C1, C2, and D were prepared on crystals from the same growth batch with the same growth conditions so it is unlikely that junctions C1 and C2 would have magnetic impurities but others surfaces would not. Another factor that will affect the Josephson coupling is the mechanical strain. The mechanical strain encountered in this experiment is probably not large enough to alter the pairing symmetry of UTe_2. Therefore, the absence of the Josephson coupling in samples C1 and C2 should be due to an intrinsic reason, the pairing symmetry of UTe_2.

4. Magnetic field dependence of the Josephson current

A hallmark of the Josephson effect is that the Josephson current or the junction resistance measured above the critical current is sensitive to the magnetic field. As shown in figs. S9 and S10, this is indeed the case even though the variation of the critical current and the junction resistance follow neither a Fraunhofer pattern as expected from a single junction of a periodic function of magnetic field for a SQUID. It should be noted that the Josephson current under a magnetic field does not necessarily follow the Fraunhofer pattern. When the junction is not sufficiently uniform, the Josephson current will respond to the magnetic field without following the Fraunhofer pattern or a periodic function that can result from the presence of two areas in the junction that features Josephson coupling.

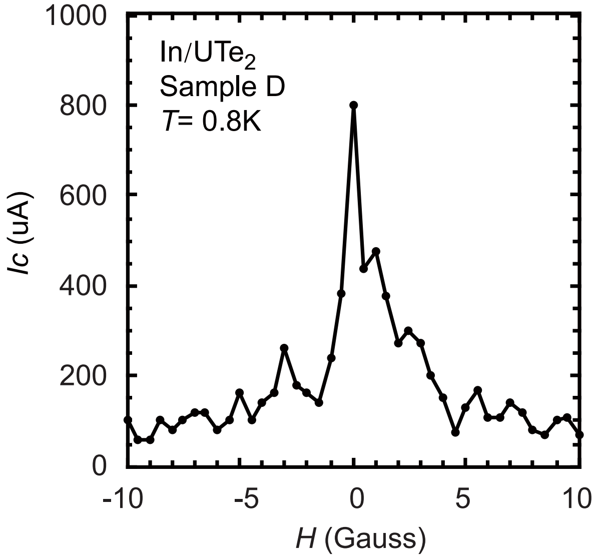

Fig. S9. Response of Josephson current to a magnetic field. Critical current (I_c) *vs.* magnetic field (H) for sample D at 0.8 K.

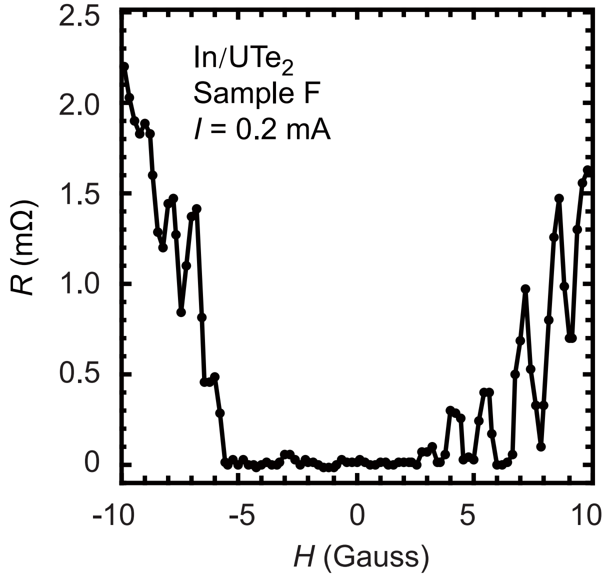

Fig. S10. Magnetic field dependence of the junction resistance Additional data on the junction resistance (R) *vs.* magnetic field (H) curves for Sample F. The measurement current I_m for sample F is 200 μA. The orientation of the junction plane for this sample was not determined.

5. Quasiparticle tunneling

We show the I $vs.$ V curves over different ranges of current for sample C2 in figs. S11A and B for completeness. The full tunneling spectra for samples C1 and C2 without the normal-state contribution subtracted are shown in figs. S11C and D. The tunneling spectra for both junctions were found to change abruptly when UTe_2 becomes superconducting, which is taken as an indication that the change is actually due to the formation of ASBS originating from the unconventional superconductivity.

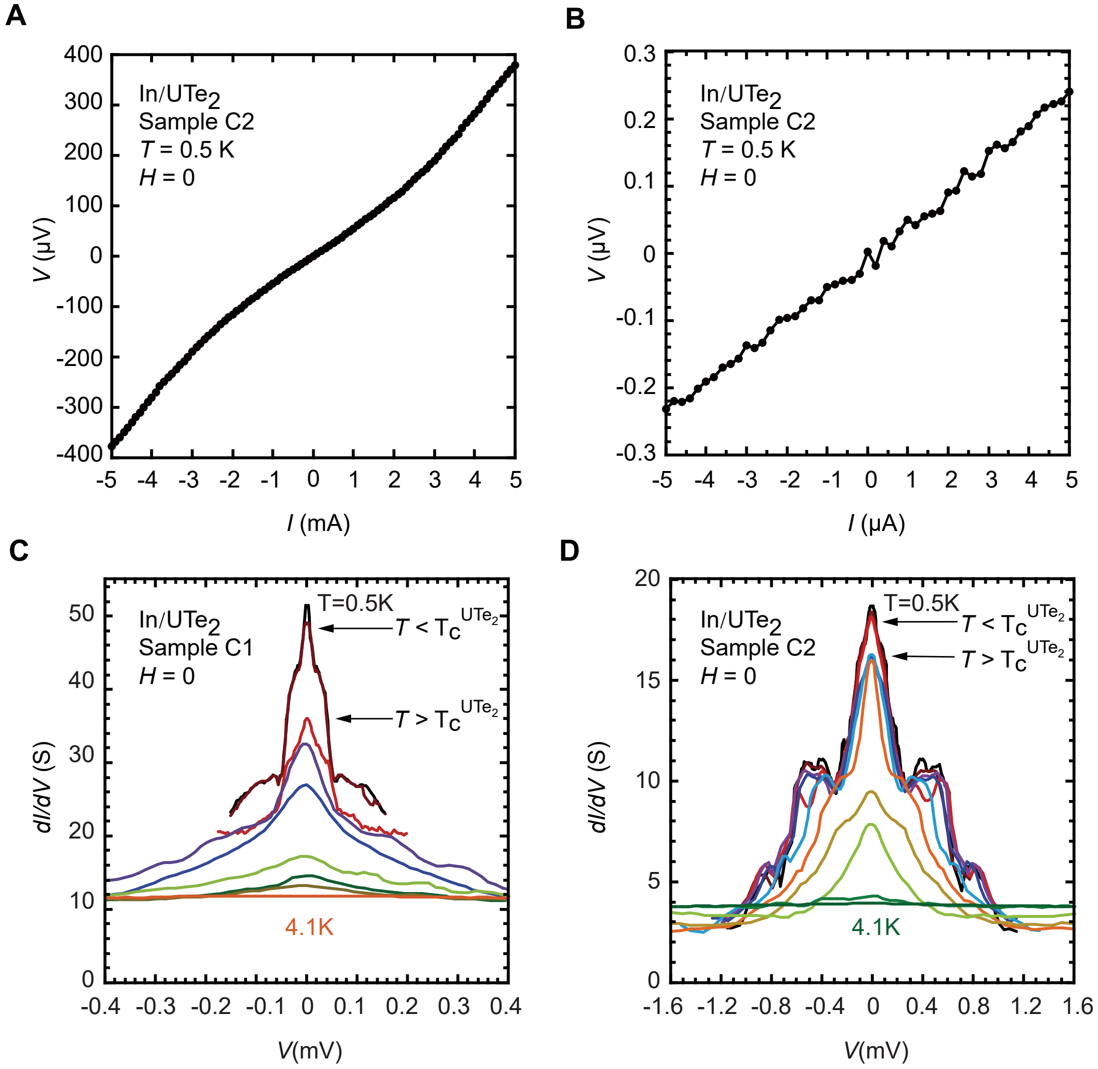

Fig. S11. Quasiparticle tunneling spectra. (A-B) Voltage (V) $vs.$ current (I) curves for sample C2 at $T = 0.5$ K measured in two ranges of current. No Josephson coupling marked by zero-voltage current is seen down to the smallest measurement currents. Sample C2 was prepared on the same crystal surface as sample C1. (C) Values of $\mathrm{d}I/\mathrm{d}V$ $vs.$ V for sample C1 at $T = 0.5$, 0.9, 2.0, 2.5, 3.0, 3.5, 3.8, 3.9, and 4.1 K. (D) Values of $\mathrm{d}I/\mathrm{d}V$ $vs.$ V for sample C2 at $T = 1.0$, 1.2, 1.4, 1.6, 2.0, 2.5, 3.0, 3.5, 3.8, 4.0, and 4.1 K. The tunneling spectra are seen to become flat at $T = 4.1$ K. A distinct jump in the junction conductance is seen when the temperature is raised across T_c.

References

1. DR Boehme, MC Nichols, RL Snyder, DP Matheis, An investigation of the tellurium-rich uranium tellurides using x-ray powder diffraction. *J. Alloy. Compd.* **179**, 37–59 (1992).
2. LP Cairns, CR Stevens, CD O'Neill, A Huxley, Composition dependence of the superconducting properties of UTe_2. *J. Physics: Condens. Matter* **32**, 415602 (2020).
3. PFS Rosa, et al., Single thermodynamic transition at 2 K in superconducting UTe_2 single crystals. *Commun. Mater.* **3**, 33 (2022).
4. Y Haga, et al., Effect of uranium deficiency on normal and superconducting properties in unconventional superconductor UTe_2. *J. Physics: Condens. Matter* **34**, 175601 (2022).
